# Did past economic prosperity affect the health related quality of life predictors? A longitudinal study on a representative sample of Slovenian family medicine patients

**DOI:** 10.1186/1471-2458-13-1160

**Published:** 2013-12-10

**Authors:** Anja Cerne, Igor Svab, Janko Kersnik, Polona Selic

**Affiliations:** 1 Department of Family Medicine, Faculty of Medicine, University of Ljubljana, Poljanski nasip 58, Ljubljana, Slovenia

**Keywords:** Quality of life, Family medicine, Psychosocial determinants, Depression, Stress, Anxiety

## Abstract

**Background:**

Health related quality of life (HRQOL) as an important measure of medical outcomes has been shown to be associated with demographic factors and the most common mental and chronic somatic diseases. This study’s aim was to identify factors predicting changes in HRQOL over a follow-up period in a representative sample of Slovenian family medicine patients.

**Methods:**

In a longitudinal multi-centred study between 2003 and 2005, data were collected from 1118 consecutive attendees from 60 family medicine practices in Slovenia on quality of life, socio-demographic factors and the presence of mental disorders, with follow-up after 6 and 24 months. Retrospective information on chronic diseases was obtained from patients` health records. In three time-sequential multiple linear regression models, data on 601 patients (53.8%) was analysed to determine factors associated with each component score of quality of life.

**Results:**

At baseline the patients were 48.58 (SE = 0.58) years of age, over half were women (386 (64.2%)) and most were Slovenian (548 (91.2%)). Quality of life was seen to improve over the two-year period. Factors significantly and consistently associated with a better mental component score of quality of life were social support, satisfactory circumstances in patients` household and absence of anxiety. Major life events in the past year and depression were shown to be risk factors for mental and physical components, while level of education, absence of long-term disability and chronic pain were identified as predictors of the physical component.

**Conclusions:**

Detection and successful treatment of depression and anxiety has a potential to lead to improved quality of life in family medicine attendees; family physicians should be alert for the early onset of these conditions, knowing that symptoms of chronic pain, depression and anxiety often overlap in patients. Poorly educated patients and those lacking social support and/or satisfactory household circumstances should be recognised and empowered, and appropriate coping mechanisms should be introduced.

## Background

Nowadays, family physicians encounter more patients suffering from more than one chronic condition than they did in the past [1]. Since a cure for such patients is often not possible, an important goal of medical practice has become improving the patients` quality of life (QOL) [2]. QOL is an individual’s perception of their position in life in the context of the culture and value system in which they live, related to their goals, expectations, standards and concerns [3,4]. As an outcome measure it has been increasingly used to evaluate the outcomes of patients with chronic diseases [5,6]. Health-related quality of life (HRQOL) has been shown to be even more suitable for use in medicine, as it focuses on health related expectations. Generally, HRQOL is interpreted as the impact that health conditions and symptoms have on an individual’s quality of life [7]. Given that, components of HRQOL are physical functioning, mental health, physical pain, general health, vitality, and social functioning [8,9].

A substantial body of research has paid interest to various diseases which could be associated with HRQOL. Common mental disorders such as affective and anxiety disorders cause distress and disability in several domains of life, and consequently have a significant effect on HRQOL [10-14]. Studies in different patient populations have also found patients diagnosed with one or more somatic diseases [15], those with multiple diseases [16], or those with self-reported long-standing illness [17] to have a lower HRQOL than the general population. HRQOL is inversely related to the number of diseases in a patient [18]. As expected, patients with diseases that produce more symptoms, e.g. osteoarthritis, congestive heart failure, and chronic obstructive pulmonary disease, have lower levels of HRQOL [1]. Aside from disease, certain demographic and social characteristics of the patient are also associated with HRQOL, i.e. a higher age [15], female gender [19,20], lower educational level, unemployment and lower income [21-23].

Since most of the studies that have been performed so far were cross-sectional, little is known about changes in HRQOL over several years, or about factors predicting a change in HRQOL. The aim of this study was to identify the factors which predict a change in HRQOL over a longer time interval.

## Methods

### Procedure and participants

In 2003, family physicians (general practitioners (GPs)) in 60 family medicine practices in Slovenia each recruited 10–20 consecutive patients, collected data, and reviewed their status after 6 and 24 months. The participating family medicine practices were selected from both urban and rural settings, and served a population with diverse socio-economic and ethnic characteristics [24]. The inclusion criterion for the patients was age 18–75 years; the exclusion criteria were poor knowledge of Slovene, a major mental disorder, or terminal disease. Patient consent was obtained and appropriate steps were taken to maintain patient confidentiality.

In 2009 additional data on chronic somatic diseases were gathered from the patients` records for the time period from 2003 to 2009, so that multi-morbidity and its patterns in these patients could be assessed and analysed. The GPs were asked to review the medical records of the subjects who were included in the original prospective cohort study on depression [24,25] and to note the presence of any of the somatic diseases included in the multi-morbidity questionnaire.

**Figure 1:**
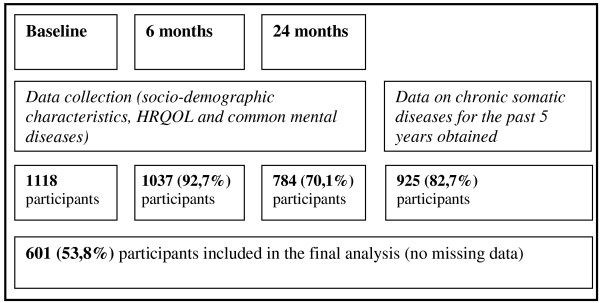
The study flowchart.

Of 1388 invited patients, 1118 agreed to participate, an initial response rate of 80.5%; the main reason for refusal was lack of time. There were no statistically significant differences in gender and age between people who were and those who were not willing to enter the study. After 6 and 24 months, there were 1037 (92.7%) and 784 (70.1%) participating patients, respectively. In the multi-morbidity part of the trial in 2009, data was collected for 925 (82.7%) of the initially recruited patients. The study flowchart is presented in Figure 1.

After excluding questionnaires with missing data, 601 questionnaires (53.8%) were analysed for this study. There were no statistically significant differences in any of the analysed variables of the initial cohort, aside from gender (female 63.4% *vs*. 64.2%, p < 0.05).

The National Medical Ethics Committee of the Republic of Slovenia approved the protocols of the studies *Predicting the Occurrence of Depression in Primary Health Care* and *The Acceptability of a Multi-factor Rating Scale for Predicting Depression in Family Medicine*.

### Measures

Information on the socio-demographic characteristics of the participants was collected using standardized questionnaires [24]. Overall health status and HRQOL was assessed by the SF-12 questionnaire [26], resulting in a Mental Component Score (MSC) and Physical Component Score (PSC), which were both defined as dependent variables in this study. Mood was examined using the Depression Section of the Composite International Diagnostic Interview (CIDI) [27], which provided psychiatric diagnoses based on symptoms experienced in the last six months, according to the Diagnostic and Statistical Manual of Mental Disorders (DSM-IV) criteria. Anxiety disorders were examined using the Patient Health Questionnaire (PHQ) [28], a brief questionnaire designed to assess DSM-IV, other anxiety disorders and panic disorder. Alcohol use was assessed by the World Health Organization Alcohol Use Disorders Identification Test (AUDIT) [29]. All these instruments were adapted for the Slovenian population in the previous research [24,25].

The multi-morbidity questionnaire covered most of the common chronic diseases and health problems in the Slovenian adult population [30], i.e. ischemic stroke, dementia, Parkinson's disease, epilepsy, coronary heart disease, malignant disease, chronic pulmonary disease, hypertension, arthritis, poor vision or hearing, chronic pain, chronic bowel disease, ulcers, gastritis, GERD, dysphagia, injury, incontinence or prostate problems, hypo- or hyperthyroidism, hypo- or hyperparathyroidism, hypo- or hyperadrenalism, porphyry and uraemia.

### Data analysis

The sample data were presented by frequency and percentage distribution for categorical variables, and by a mean value and standard deviation for continuous variables. In general, the MCS and PCS of the SF-12 were computed by a linear weighting of the 12 items in the questionnaire. In univariate analyses the associations (i.e. mean comparisons) between the independent variables and the MCS and PCS of HRQOL were calculated. Variables which proved statistically significant on at least two out of three data collections or which had been proven to be associated with MCS and/or PCS in a known body of research were used in multivariable linear regression modelling. A linear regression was conducted to T_0_, T_6_ and T_24_ sequentially and separately. Multivariate models were performed separately for the MCS and PCS components of HRQOL at the first data collection, after 6 and after 24 months (T_0_, T_6_ or T_24_), aiming to determine the impact of time on each component of the quality of life. Independent variables included in the modelling process predicting MCS are shown in Table 1, while those predicting PCS are presented in Table 2. Statistical significance was set at p < 0.05. The analysis was performed using the statistical package SPSS, version 13.0 (SPSS for Windows, Chicago: SPSS Inc.).

**Table 1:** Comparison of β-coefficients at baseline, after 6 and 24 months: Predictors of better mental component score of HRQOL

**Independent variables**	**β (601)**	**β (601)**	**β (601)**
	**0 months**	**6 months**	**24 months**
Better PCS	**−0.086**	−0.041	**−0.134**
Higher age	0.065	**0.099**	**0.077**
Male gender	**0.105**	0.072	**0.100**
Higher level of education	0.006	−0.060	−0.045
Better financial status	**0.097**	0.059	**0.088**
**Better circumstances in a household**	**0.127**	**0.175**	**0.091**
**Better social support**	**0.159**	**0.133**	**0.099**
**Stressful life events – present**	**−0.071**	**−0.129**	**−0.082**
Abuse in childhood – present	−0.015	−0.057	−0.064
Discrimination – present	−0.066	0.020	0.002
Relatives with problems – present	−0.006	−0.025	−0.028
**Depression – present**	**−0.346**	**−0.263**	**−0.386**
Higher AUDIT score	−0.006	0.004	0.031
**Anxiety disorders – present**	**−0.196**	**−0.214**	**−0.167**
Higher number of chronic diseases	−0.015	0.023	−0.054

PCS – physical component score; MCS – mental component score; bold: p ≤ 0.05.

Bolded independent variables were statistically significant in all three sequences.

*F*_*0*_ *= 24.06, p*_*0*_ *< 0.001; F*_*6*_ *= 23.32; p*_*6*_ *< 0.001; F*_*24*_ *= 21.05; p*_*24*_ *< 0.001.*

**Table 2:** Comparison of β-coefficients at baseline, after 6 and 24 months: Predictors of better physical component score of HRQOL

**Independent variables**	**β (601)**	**β (601)**	**β (601)**
	**0 months (T**_**0**_**)**	**6 months (T**_**6**_**)**	**24 months (T**_**24**_**)**
Better MCS	**−0.103**	−0.065	**−0.126**
Higher age	**−0.140**	**−0.197**	−0.071
Male gender	0.053	**0.098**	**0.085**
**Higher level of education**	**0.140**	**0.124**	**0.096**
Better financial status	**0.102**	0.047	0.049
Unemployed	0.039	0.059	−0.026
Retired	0.055	0.040	0.023
Better circumstances in a household	0.035	0.019	0.021
Better social support	0.050	**0.080**	0.051
**Stressful life events – present**	**−0.115**	**−0.107**	**−0.068**
**Self-rated health**	**−0.360**	**−0.415**	**−0.433**
**Depression – present**	**−0.094**	**−0.101**	**−0.186**
Higher AUDIT score	0.027	0.018	0.009
Anxiety disorders – present	0.005	−0.039	**−0.093**
Higher number of chronic diseases	−0.053	0.031	0.080
Diabetes mellitus - present	< 0.001	−0.012	−0.006
Coronary heart disease – present	−0.026	−0.045	−0.060
Chronic pulmonary disease – present	−0.002	0.009	**−0.067**
Arterial hypertension - present	0.098	0.063	−0.013
Arthritis – present	−0.030	−0.042	**−0.087**
**Chronic pain – present**	**−0.082**	**−0.141**	**−0.169**

PCS – physical component score; MCS – mental component score; bold: p ≤ 0.05.

Bolded independent variables were statistically significant in all three sequences.

*F*_*0*_ *= 13.57, p*_*0*_ *< 0.001; F*_*6*_ *= 20.75; p*_*6*_ *< 0.001; F*_*24*_ *= 23.87; p*_*24*_ *< 0.001.*

## Results

At baseline, the participants were 48.58 (SE = 0.58) years of age, more than half were women (386 (64.2%)), and the majority were of Slovenian origin (548 (91.2%)). Most had average education (367 (61.1%) finished high school and 102 (17.0%) achieved college or more) and did not have financial problems (509 (84.7%) doing ‘all right’ or better). More demographic characteristics for the period of the study are presented in Table 3.

**Table 3:** Demographic characteristics of patients for the study period

		**N (%)**		
		**0 months**	**6 months**	**24 months**
**Gender**	Male	215 (35.8%)	215 (35.8%)	215 (35.8%)
	Female	386 (64.2%)	386 (64.2%)	386 (64.2%)
**Nationality**	Slovenian	548 (91.2%)	544 (90.5%)	592 (98.5%)
	Other	53 (8.8%)	57 (9.5%)	9 (1.5%)
**Religiosity**	Yes	374 (62.2%)	389 (64.7%)	408 (67.9%)
	No	227 (38.8%)	212 (35.3%)	193 (32.1%)
**Employment status**	Employed	332 (53.6%)	363 (60.4%)	357 (59.4%)
	Unemployed, student	60 (10.0%)	62 (10.3%)	49 (8.2%)
	Retired	216 (35.9%)	175 (29.1%)	195 (32.4%)
**Educational level**	Up to elementary school	132 (22.0%)	122 (20.3%)	117 (19.5%)
	High school	367 (61.1%)	375 (62.4%)	369 (61.4%)
	College and higher	102 (17.0%)	104 (17.3%)	112 (18.6%)
**Self-rated financial status**	Major financial problems	15 (2.5%)	10 (1.7%)	12 (2.0%)
	Minor financial problems	77 (12.8%)	76 (12.6%)	71 (11.8%)
	Comfortable living	473 (78.7%)	480 (79.9%)	474 (78.9%)
	Very comfortable living	36 (6.0%)	35 (5.8%)	44 (7.3%)
**Marital status**	Married or living with partner	423 (70.4%)	422 (70.2%)	416 (69.2%)
	Single	175 (29.1%)	176 (29.3%)	181 (30.1%)

**Figure 2:**
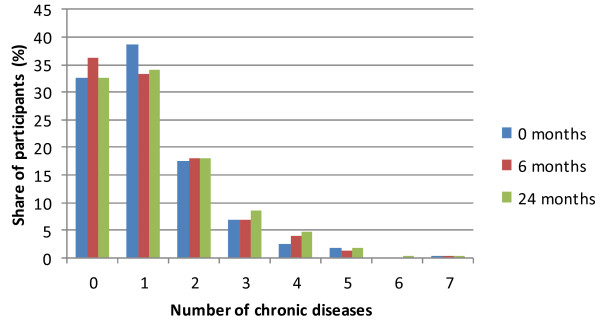
Multi-morbidity in patients for the study period.

The social determinants of the patients in the period of the study are shown in Table 4. Of all the participants, 309 (51.4%) had experienced at least one stressful life event in the past year. The average value of the estimated social support of relatives and friends on a scale from 7 to 21 was 18.8 (18.6-19.0), and the average satisfaction with the patients’ own household on a scale from 1 to 5 was 4.3 (4.2-4.3). Of all, 93 (15.5%) participants were diagnosed depressive, and 49 (8.2%) were diagnosed with anxiety disorders at baseline. Just over two thirds of the patients (405 (67.4%)) had at least one chronic somatic disease, and about one third (173 (28.8%)) of the patients were multi-morbid at baseline. Over the time of the study there was a rise in the average number of chronic conditions per patient (from 1.14 to 1.26) and in the percentage of multi-morbid patients (from 28.8% to 33.4%), which was to be expected due to ageing. The multi-morbidity of the patients for the study period is presented in Figure 2.

**Table 4:** Social determinants of patients for the study period

		**N (%)**		
		**0 months**	**6 months**	**24 months**
**Household**	Living alone	64 (10.6%)	62 (10.3%)	63 (10.5%)
	Living with someone	536 (89.2%)	536 (89.2%)	538 (89.5%)
**Stressful life events**	Present	309 (51.4%)	259 (43.1%)	292 (48.6%)
**Abuses in childhood**	Present	126 (21.0%)	89 (14.8%)	100 (16.6%)
**Discrimination**	Present	46 (7.7%)	32 (5.3%)	29 (4.8%)
**Relatives with problems**	Present	114 (19.0%)	97 (16.1%)	97 (16.1%)
**Circumstances in a household**	M (95% confidence interval) 1–5 (5 = very satisfactory)	4.28 (4.21- 4.34)	4.30 (4.24-4.37)	4.32 (4.26-4.39)
**Social support of family and friends**	M (95% confidence interval) 7–21 (21 = excellent personal relationships)	18.77 (18.55-18.99)	19.01 (18.78-19.23)	19.34 (19.12-19.55)

Legend: M - mean.

At the time of inclusion in the study, 317 (52.7%) of patients reported long term disease or disability, which was a criteria for self-rated health; this number had dropped to 277 (46.1%) at the two year follow-up. The most frequent chronic condition reported was arterial hypertension in 165 patients (27.5%), followed by chronic pain (n = 122 (20.3%)). Self-rated health and health conditions for the study period are presented in Table 5.

**Table 5:** Self-rated health, presence of chronic somatic and mental diseases in patients for the study period

		**N (%)**		
		**0 months**	**6 months**	**24 months**
**Self-rated health**		317 (52.7%)	294 (48.9%)	277 (46.1%)
**Arterial hypertension**		165 (27.5%)	173 (28.8%)	178 (29.6%)
**Chronic pain**		122 (20.3%)	118 (19.6%)	127 (21.1%)
**Injury**		82 (13.6%)	62 (10.3%)	72 (12.0%)
**Gastric ulcer**		53 (8.8%)	52 (8.7%)	60 (10.0%)
**Artritis**		39 (6.5%)	43 (7.2%)	53 (8.8%)
**Diabetes mellitus**		36 (6.0%)	39 (6.5%)	39 (6.5%)
**Coronary heart disease**		35 (5.8%)	36 (6.0%)	39 (6.5%)
**Chronic pulmonary disease**		33 (5.5%)	30 (5.0%)	34 (5.7%)
**Deafness**		30 (5.0%)	34 (5.7%)	42 (7.0%)
**Presence of chronic somatic diseases**		405 (67.4%)	383 (63.7%)	405 (67.4%)
**Depression**		93 (15.5%)	69 (11.5%)	72 (12.0%)
**Anxiety disorders**		49 (8.2%)	39 (6.5%)	40 (6.7%)
**Alcohol consumption**	Lower risk drinking	571 (95.0%)	580 (96.5%)	591 (98.3%)
	Risk drinking	30 (5.0%)	21 (3.5%)	10 (1.7%)

### Predictors of improved mental component score of HRQOL

The mean MCS at baseline was 49.80 (48.93-50.67) and increased to 53.14 (52.37-53.91); the mean PCS was 42.28 (41.41-43.15) and increased to 44.68 (43.82-45.55) during the two year follow-up.

The multivariate modelling was performed sequentially and separately for the first (T_0_), second (T_6_) and third (T24) data collection period (F_0_ = 24.06, p_0_ < 0.001; F_6_ = 23.32; p_6_ < 0.001; F_24_ = 21.05; p_24_ < 0.001) explaining 38.2%, 37.4% and 35.2% of the variance respectively.

Higher values on the MCS component of HRQOL were associated with social determinants in all three models, i.e. better social support from friends and relatives (β_0_ = 0.159, p_0_ ≤ 0.001, β_6_ = 0.133, p_6_ ≤ 0.001, β_24_ = 0.099, p_24_ = 0.006), absence of significant life events in the past 12 months (β_0_ = −0.071, p_0_ = 0.040, β_6_ = −0.129, p_6_ ≤ 0.001, β_24_ = −0.082, p_24_ = 0.018), satisfactory circumstances in the household (β_0_ = 0.127, p_0_ ≤ 0.001, β_6_ = 0.175, p_6_ < 0.001, β_24_ = 0.091, p_24_ = 0.014), and with absence of anxiety (β_0_ = 0.196, p_0_ ≤ 0.001, β_6_ = −0.214, p_6_ ≤ 0.001, β_24_ = −0.167, p_24_ ≤ 0.001) or depression (β_0_ = 0.346, p_0_ ≤ 0.001, β_6_ = −0.263, p_6_ ≤ 0.001, β_24=_–0.386, p_24_ ≤ 0.001). Predictors of better MCS are presented in Table 1.

### Predictors of better physical component score of HRQOL

A linear regression was conducted for T_0_, T_6_ and T_24_ sequentially and separately (F_0_ = 13.57, p_0_ < 0.001; F_6_ = 20.75; p_6_ < 0.001; F_24_ = 23.87; p_24_ < 0.001), 33.0%, 42.9% and 46.5% of the variance respectively were explained.

Higher values on the PCS component of HRQOL were in all three models associated with higher educational attainment (β_0_ = 0.140, p_0_ ≤ 0.001, β_6_ = 0.124, p_6_ ≤ 0.001, β_24_ = 0.096, p_24_ = 0.005), absence of chronic pain (β_0_ = −0.082, p_0_ = 0.049, β_6_ = −0.141, p_6_ ≤ 0.001, β_24_ = −0.169, p_24_ ≤ 0.001), depression (β_0_ = −0.094, p_0_ = 0.019, β_6_ = −0.101, p_6_ = 0.008, β_24_ = −0.186, p_24_ ≤ 0.001) and better health self-evaluation (β_0_ = −0.360, p_0≤_0.001, β_6_ = −0.415, p_6_ ≤ 0.001, β_24_ = −0.433, p_24_ ≤ 0.001) together with absence of stressful life events (β_0_ = −0.115, p_0_ ≤ 0.001, β_6_ = −0.107, p_6_ ≤ 0.001, β_24_ = −0.068, p_24_ = 0.034) which is shown in Table 2.

## Discussion

The presented findings in a prospective cohort of family medicine patients showed a statistically significant change in the mental and physical components of wellbeing at follow-up. Quality of life improved significantly in better educated people with good social support and satisfactory circumstances in their household, the absence of major life events in the past 12 months, good self-assessment of health, and the absence of depression, anxiety disorders and chronic pain (Tables 1 and 2). The results of this longitudinal study expanded the previous evidence from cross-sectional population-based and clinical studies that investigated the association of several variables with HRQOL.

### A change in the mental and physical components of health related quality of life during the follow-up period

The rise in MCS and PCS is not in accordance with the previously published body of research [31-34], where a decline in HRQOL over time was shown. However, most of the longitudinal studies have found the MCS remaining relatively stable over time or even improving, while PCS declined with time [35-38]. Longitudinal studies with a longer observation period have shown declines in HRQOL domains. A possible explanation of both PCS and MCS improving over time could lie with the socio-economic context of the country during the data collection period. From 2003 to approximately 2008, there was political stability and economic growth in Slovenia [39]; the country entered the European Union and adopted the euro as the national currency. The increase in HRQOL could therefore be explained by the positive socio-economic situation in the country and the subjective assessment of the HRQOL. It is also likely that it was too short a time period to detect changes in HRQOL due to ageing, which is known to be associated with a lower HRQOL [15,21,34]. Since the average participant was middle-aged, apparently no severe health deteriorations occurred during the study period. Finally, the cohort could have received more attention from their physicians; the simple fact that the patient was included in this longitudinal study could have ensured that they were better taken care of. As this would have introduced an additional variable, and would have interfered with the real-life approach of the study, the participating GPs did not receive any specific additional education about HRQOL and its dimensions, or any training in the diagnosis of the health conditions in question. However, the GPs were aware of the patients` voluntary participation in the study, so a Hawthorne effect could have appeared.

### Socio-demographic predictors of health related quality of life

One socio-demographic characteristic, i.e. higher educational attainment, was identified as being associated with better PCS (Table 2), which is in line with other studies [21]. It is worth mentioning that in Slovenia the consultation time for higher-educated patients has been shown to be longer than for less well-educated patients [40]; getting more attention and medical information or guidance could affect HRQL. Aside from the amount of given advice, level of education was identified as a relevant predictor of recall in Slovenian family medicine patients, although level of education alone does not reflect memory potential [41].

A better MCS component of HRQOL was associated with better social support and the absence of significant life events (Table 1), and the same pattern was shown to be associated with better PCS (Table 2). Good social support was shown to be a protective factor [42,43], while significant life events as stressors were shown to be associated with a lower MCS component of HRQOL, concordant with other findings [44-46].

### Health predictors of health related quality of life

Depression was associated with the MCS as well as the PCS component of HRQOL (Tables 1 and 2), while anxiety was associated only with the MCS component of HRQOL (Table 1). Mental health problems are considered as even more important than physical problems with regard to quality of life perception and assessment [47-49]. Self-rated health has been shown to be highly associated with the PCS component of HRQL, as also found by other authors [50,51]. Aside from chronic pain being associated with the PCS component of HRQOL (Table 2), concordant with findings of other researchers [52-56], we did not find statistically significant associations between chronic diseases and either the PCS or MCS component of HRQOL as others did [22,57]. A cross-sectional study performed in 2011 in Slovenia showed a significantly higher prevalence of chronic diseases in those with a lower socio-economic status and in pensioners [58]. Since level of education has been shown to be associated with socioeconomic status in the Slovenian active population [59], the explanation could lie partially in this characteristic. Selic and co-workers [60] reported the burden of somatic co-morbidity to be smaller than the impact of psychosocial determinants when identifying the patterns of physical co-morbidity and factors associated with the onset of depression. Psychosocial determinants, i.e. the feeling of safety at home and the absence of problems in intimate relationships, were interpreted as protective factors [60]. On the other hand, in a longitudinal study of the predictors of HRQOL in patients with arterial hypertension, Maatouk and colleagues reported most of the somatic diseases to be associated with a lower PCS after five years [61]. Due to the quality of data collected, in this study the impact of neither the severity of chronic somatic disease nor the time since diagnosis could be shown, although these are known to have a particular impact on HRQOL according to patients’ adaptation to disease, as stated by Schwartz and Sprangers in the response shift theory [62,63]. We embrace the explanation that individuals experiencing a change in their health status may also change their appraisals, internal standards and values with regard to QOL and will try to further research possible associations.

In the multivariate modelling conducted for T_0_, T_6_ and T_24_ sequentially and separately, 38.2%, 37.4% and 35.2% of the variance respectively were explained for the MCS and 33.0%, 42.9% and 46.5% of the variance respectively were explained for the PCS of HRQL. The strength of identified predictors for PCS is therefore greater compared to predictors of MCS, although the absence of depression and stressful life events in the previous year acted as the most important predictors of HRQL as a whole. Further research is needed to be able to explain more of the total variance, also considering that this study was limited by its reliance on self-reported data (questionnaires on quality of life, depression, anxiety disorders and alcohol consumption), which raises questions about the potential for method variance (i.e. same-source measurement bias) to account for our findings. Although the phenomena being studied could have been assessed only by questionnaire administration, asking respondents to report an internal state or perception, it would be useful in the design of further research if some other measures were incorporated to mitigate the potential effects of method variance.

### Strengths and limitations of the study

One of the major strengths of our study is longitudinal follow-up of the patient cohort providing three consecutive measurements of HRQOL and the variables associated with it. Moreover, the study was performed on a representative sample of family medicine patients. The data about patients’ chronic diseases were obtained from their medical records, which is more accurate than patients’ self reports.

Aside from the reliance on mostly self-reported data, a possible limitation to the study could be the missing data, since only about half of the original cohort was included in the analysis. However, given that there were no statistically significant differences in any of the analysed variables with the initial cohort except for gender, we believe that the initially planned representativeness of the sample was not lost.

In spite of the large sample size, the prevalence of depression (93 (15.5%)), anxiety disorder (49 (8.2%)) and harmful alcohol drinking (30 (5.0%)) (Table 5) was lower than in previous research in Slovenia [30,60] and resulted in a low number of subjects with common mental disorders. In comparison to a representative sample of Slovenian general practice attendees, described by Svab and co-workers [58], the patients in our sample were younger (at baseline 48.6 and after 24 months 50.4 *vs.* 51.7 years of age), better educated (primary school 22% *vs.* 41%; high school 62% *vs.* 48%; college or university degree 17% *vs.* 11%), with a higher proportion of women (64.2% *vs.* 54.8%). Aside from the latter, all the above-mentioned characteristics of the sample were apparently in favour of this lower prevalence; nevertheless, further research should be carried out to validate the prevalence data.

Furthermore, only information of the presence or absence of chronic diseases was obtained, without data on their severity or complications, which have been shown to be associated with HRQOL. Since the physicians were aware of the patients` voluntary participation in the study, a non-specific influence of study participation on their usual care patterns should be considered with regard to the identification of chronic diseases and their treatment in the cohort. Finally, information about the time since diagnosis was not taken into account in the analysis, but is expected to be associated with HRQOL.

## Conclusions

In order to improve HRQOL in patients, GPs should pay attention to the timely recognition and management of depression and anxiety and also chronic pain, as depression can intensify the feeling of chronic pain, while living with chronic pain can contribute to depression. The symptoms of chronic pain, depression and anxiety often overlap in patients.

Moreover, GPs should have a good insight in the social support system and satisfaction with household circumstances of their patients, and take into account education levels, both of which contribute to HRQOL. Since the level of education is marked on patients` medical charts, it would be possible for GPs to take this characteristic into consideration when providing either more complex or a larger volume of medical information, or trying to empower patients regarding HRQL. Although GPs are not able to control stressful life events affecting their patients, they can explore patients` coping mechanisms and provide information about active, rather than passive, coping strategies.

## Abbreviations

QOL: Quality of life; HRQOL: Health-related quality of life; MCS: Mental component score; PCS: Physical component score; CIDI: Depression section of the composite international diagnostic interview; DSM-IV: Diagnostic and statistical manual of mental disorders; PHQ: Patient health questionnaire; AUDIT: Alcohol use disorders identification test.

## Competing interests

The authors declare that they have no competing interests.

## Authors’ contributions

AC participated in the study execution, contributed to the data analysis and participated in drafting the manuscript. IS conceived the study and participated in the data interpretation. JK participated in data collection and interpretation. PS participated in the design of the study, carried out the coordination of the study, and drafted the manuscript. All the authors read and approved the final manuscript.

## Authors’ information

AC: PhD Family Medicine, Junior Researcher at the Department of Family Medicine.

IS: PhD Family Medicine, Professor, Head of the Department of Family Medicine.

JK: PhD Family Medicine, Professor, Head of the Research Division at the Department of Family Medicine.

PS: PhD Clinical Psychology, Senior Researcher and Assistant Professor at the Department of Family Medicine.

## Pre-publication history

The pre-publication history for this paper can be accessed here:

http://www.biomedcentral.com/1471-2458/13/1160/prepub
